# The Arcuate Nucleus of the Hypothalamus and Metabolic Regulation: An Emerging Role for Renin–Angiotensin Pathways

**DOI:** 10.3390/ijms22137050

**Published:** 2021-06-30

**Authors:** Darren Mehay, Yuval Silberman, Amy C. Arnold

**Affiliations:** Department of Neural and Behavioral Sciences, Pennsylvania State University College of Medicine, Hershey, PA 17033, USA; dmehay@pennstatehealth.psu.edu (D.M.); ysilberman@pennstatehealth.psu.edu (Y.S.)

**Keywords:** angiotensin, obesity, energy balance, neurocircuitry, cardiovascular

## Abstract

Obesity is a chronic state of energy imbalance that represents a major public health problem and greatly increases the risk for developing hypertension, hyperglycemia, and a multitude of related pathologies that encompass the metabolic syndrome. The underlying mechanisms and optimal treatment strategies for obesity, however, are still not fully understood. The control of energy balance involves the actions of circulating hormones on a widely distributed network of brain regions involved in the regulation of food intake and energy expenditure, including the arcuate nucleus of the hypothalamus. While obesity is known to disrupt neurocircuits controlling energy balance, including those in the hypothalamic arcuate nucleus, the pharmacological targeting of these central mechanisms often produces adverse cardiovascular and other off-target effects. This highlights the critical need to identify new anti-obesity drugs that can activate central neurocircuits to induce weight loss without negatively impacting blood pressure control. The renin–angiotensin system may provide this ideal target, as recent studies show this hormonal system can engage neurocircuits originating in the arcuate nucleus to improve energy balance without elevating blood pressure in animal models. This review will summarize the current knowledge of renin–angiotensin system actions within the arcuate nucleus for control of energy balance, with a focus on emerging roles for angiotensin II, prorenin, and angiotensin-(1–7) pathways.

## 1. Introduction

Obesity is a major public health problem, with 39.8% of the United States population considered obese in 2016 [1]. Importantly, obesity greatly increases the risk for developing hypertension, hyperglycemia, and a multitude of related pathologies that encompass the metabolic syndrome [2]. In fact, the American Heart Association has cited obesity as one the biggest current challenges in improving cardiovascular health [3]. At the most fundamental level, obesity occurs when caloric intake exceeds energy expenditure to promote excess energy storage in white adipose tissue [2]. While the restoration of energy balance is expected to prevent or slow the onset of obesity, the underlying mechanisms and optimal strategies to achieve this remain poorly understood. As evidence of this, lifestyle modifications only produce modest effects on long-term weight loss [4]. Furthermore, several anti-obesity drugs, which target central pathways involved in the regulation of appetite or energy expenditure, have been withdrawn from the market due to limited efficacy and adverse cardiovascular and other off-target effects [5,6]. These findings illustrate the critical need to identify new pharmacological targets that can be utilized to promote positive metabolic changes for the treatment of obesity without adversely impacting cardiovascular control. 

The control of energy balance involves complex and integrated interactions between behavioral and physiological factors. In terms of physiological factors, a growing area of research is to better understand how peripheral hormones interact with a widely distributed network of brain circuits involved in the control of energy balance [7,8], with the ultimate goal of developing new approaches that target specific neuronal pathways to induce positive metabolic effects without eliciting adverse cardiovascular effects. In this regard, the arcuate nucleus of the hypothalamus (ARC) has emerged as an important brain region due to its ability to sense circulating hormones and to modulate neural pathways controlling food intake, energy expenditure, and blood pressure [9,10]. While still a relatively new area of investigation, recent studies suggest the renin–angiotensin system (RAS) may provide an ideal hormonal target to engage ARC neurocircuits to improve energy balance in obesity without elevating blood pressure. This review will summarize current knowledge of RAS actions within the ARC for control of energy balance, with a focus on emerging roles for angiotensin II, prorenin, and angiotensin-(1–7) pathways. Additionally, key gaps in knowledge will be identified, including sex differences in RAS effects on ARC neurocircuits, as well as the need for further integrative preclinical research to better understand how to target specific ARC neuronal subpopulations to induce weight loss while avoiding the adverse cardiovascular consequences historically seen with anti-obesity drugs.

## 2. Overview of Arcuate Neurocircuits Controlling Energy Balance

**Figure 1:**
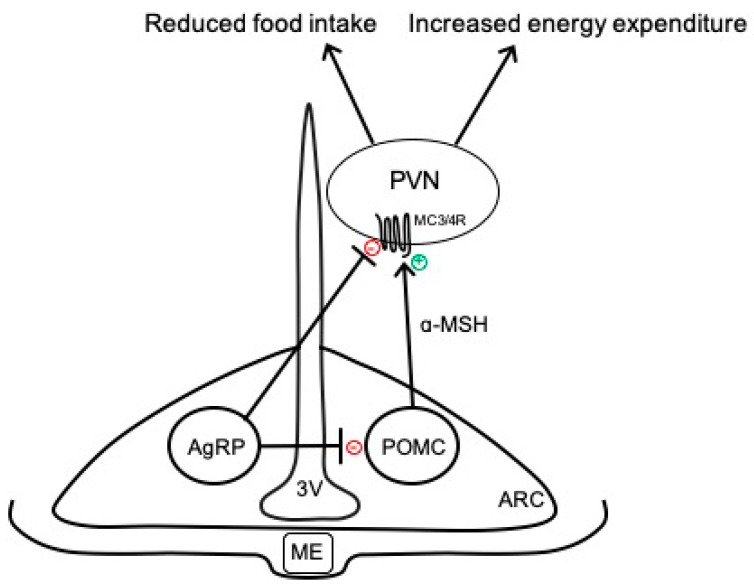
Simplified overview of classical neurocircuits within the arcuate nucleus of the hypothalamus controlling energy balance. 3V, third ventricle; AgRP, agouti-related peptide; α-MSH, α-melanocyte stimulating hormone; ARC, arcuate nucleus of the hypothalamus; MC3/4R, melanocortin-3 and -4 receptors; ME, median eminence; POMC, proopiomelanocortin; PVN, paraventricular nucleus of the hypothalamus.

The hypothalamus is a key brain region that integrates peripheral information about the nutritional status of the body and can emit an endocrine response to regulate food intake or an autonomic nervous system response to regulate energy expenditure [9]. In particular, the ARC has emerged as an important contributor to energy balance and is adjacent to the median eminence, which is blood–brain barrier permeable, thus allowing access to peripheral signals. As shown in Figure 1, there are two primary neuronal subpopulations within the ARC controlling energy balance: (1) ARC^POMC^ neurons that contain the pro-opiomelanocortin (POMC) gene, which is proteolytically cleaved to produce biologically active peptides including α-melanocyte stimulating hormone (α-MSH), adrenocorticotropic hormone, and β-endorphin; and (2) ARC^AgRP^ neurons that co-express neuropeptide Y (NPY) and agouti-related peptide (AgRP) [11]. Classically, the activation of ARC^POMC^ neurons results in the release of α-MSH, which binds to melanocortin 3 and 4 receptor (MC3/4R)-expressing neurons in the paraventricular nucleus of the hypothalamus (PVN) and brainstem to reduce food intake and modulate the autonomic nervous system pathways controlling adipose thermogenesis and energy expenditure [12,13]. The activity of these ARC^POMC^ neurons is regulated by numerous peripheral hormonal signals, including leptin, insulin, glucocorticoids, and thyroid hormones [14]. ARC^AgRP^ neurons inhibit ARC^POMC^ neurons as well as downstream melanocortin pathways to conversely increase food intake and decrease energy expenditure (Figure 1) [15].

In addition to the classically defined neurocircuits described above and in Figure 1, accumulating evidence supports the heterogeneity of ARC^POMC^ neurons in terms of responses to peripheral signals, receptors expressed, and neurochemical identity [11,16,17]. For example, separate populations of leptin- and insulin-responsive POMC neurons exist in the ARC [18]. In addition to neuropeptides, such as α-MSH, ARC^POMC^ neurons can co-release amino acid transmitters, such as glutamate and γ-aminobutyric acid (GABA), to exert more rapid physiological actions [11,17]. Indeed, functionally distinct subsets of glutamatergic and GABAergic ARC^POMC^ neurons exist in rodents, with only a small percentage co-releasing both neurotransmitters [19,20]. These neurotransmitter subpopulations have distinct localization in the ARC, receive separate hormonal and neuronal inputs, and send outputs to different brain regions [20]. ARC^AgRP^ neurons are also responsive to various stimuli, such as insulin and leptin, and are primarily GABAergic; however, subsets of these neurons express somatostatin (Sst) or corticotropin-releasing hormone [21]. The functional importance of these distinct subsets of ARC^POMC^ and ARC^AgRP^ neurons to the neural control of energy balance is not fully understood but may provide an opportunity to develop selective approaches for the targeting of metabolic versus cardiovascular outcomes.

The ability of ARC neurocircuits to modulate energy expenditure involves the sympathetic-mediated activation of thermogenesis in adipose tissue. The ARC and PVN are functionally connected to brown adipose tissue via polysynaptic intra-hypothalamic and brainstem pathways that modulate the sympathetic innervation of adipose depots [9]. The stimulation of these hypothalamic-brown adipose-thermogenic pathways results in the disinhibition of the raphe nucleus and increased sympathetic outflow through the spinal cord. This sympathetic activation results in the release of norepinephrine to activate postsynaptic β3-adrenoreceptors on adipocytes, which elicits mitochondrial uncoupling to induce thermogenesis and increase energy expenditure [9,22]. In addition to the ARC, several other nuclei contribute to sympathetic-mediated brown adipose thermogenesis (e.g., preoptic area, dorsomedial, lateral, paraventricular, raphe) [9]. While the targeting of brown adipose tissue has been of great interest for obesity treatment, this approach appears limited in efficacy due to the small amounts of this tissue in humans [23]. A more recent discovery is that white adipose tissue, which is typically involved in energy storage, also receives sympathetic input from hypothalamic pathways, including the ARC, and can be converted to function more like brown adipose tissue to promote thermogenesis [24]. This white adipose “browning” or “beiging” has emerged as a potential target in the development of innovative pharmacological strategies for the treatment of obesity. White adipose browning is a particularly attractive target given the large amounts of this tissue in obesity, and that it can be physiologically or pharmacologically stimulated (e.g., cold, β3-agonists) [25,26]. An important caveat when activating ARC-adipose pathways to induce thermogenesis and increase energy expenditure, however, is that it can produce diffuse sympathetic activation to cardiovascular end organs, resulting in elevated blood pressure. Identifying new targets that selectively increase sympathetic outflow to either brown or white adipose tissue, without affecting autonomic tone to cardiovascular organs, would be a major advance.

Obesity is well recognized to disrupt ARC neurocircuits controlling energy balance. Prolonged high-fat diet (HFD) feeding in mice induces an obese phenotype that is associated with increased neuronal activity in the hypothalamus, as measured by c-fos-like immunoreactivity [27,28]. This hypothalamic neuronal activation in response to HFD may in part reflect the increased activity of ARC^AgRP^ neurons to provide inhibitory signaling to ARC^POMC^ neurons [29]. A more recent study showed that chronic HFD in mice is associated with the suppression of leptin gene-expression signaling pathways selectively in ARC^AgRP^ neurons, which may contribute to metabolic leptin resistance and be secondary to altered cAMP response element-binding protein signaling [30]. In addition to enhanced ARC^AgRP^ activity, POMC neuronal firing rates are reduced by HFD [31]. This reduced ARC^POMC^ neuronal activity in obesity has been attributed to numerous factors, including inflammation, reactive oxygen species, endoplasmic reticulum stress, and altered hypothalamic autophagy [16]. Obesity is also associated with decreased brown adipose tissue activity and attenuated metabolic responses to the hormone leptin, which, under normal conditions, promotes satiety and stimulates sympathetic outflow to brown adipose tissue to increase resting metabolic rate [32,33]. Leptin-stimulated sympathetic outflow to cardiovascular organs, however, remains intact in obesity, suggesting a phenomenon of selective leptin resistance [33,34]. Overall, chronic HFD exposure is associated with the remodeling of hypothalamic circuits, including increased ARC^AgRP^ and decreased ARC^POMC^ neuronal activity, which can functionally contribute to energy imbalance and related metabolic complications in obesity.

## 3. Circulating Renin–Angiotensin System in Obesity

**Figure 2:**
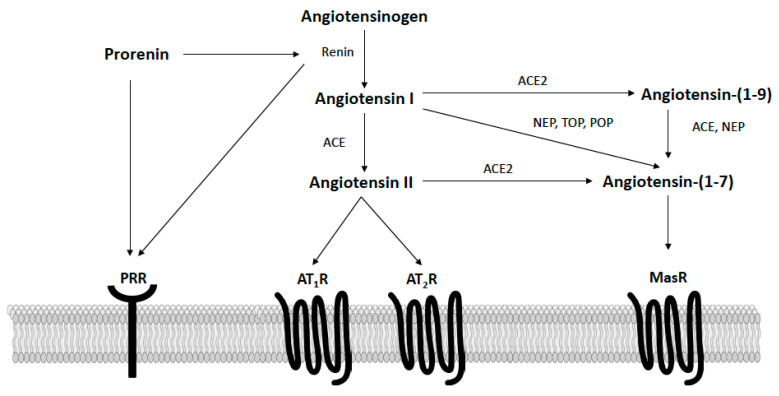
Overview of the renin–angiotensin system focusing on components known to potentially impact the control of energy balance in the arcuate nucleus of the hypothalamus. AT_1_R, angiotensin II type I receptors; AT_2_R, angiotensin II type II receptors; PRR, prorenin receptor; MasR, angiotensin-(1–7) *mas* receptors; ACE, angiotensin converting enzyme; ACE2, angiotensin converting enzyme 2; NEP, neprilysin; POP, prolyl oligopeptidase; TOP, thimet oligopeptidase.

The RAS is well recognized as an important hormonal regulator of both metabolic and cardiovascular functions in obesity. As shown in Figure 2, in the classically defined RAS, the precursor angiotensinogen is converted to angiotensin I via renin, which is then converted to angiotensin II via angiotensin-converting enzyme (ACE). Circulating and adipose angiotensin II levels are elevated in obesity and bind type I receptors (AT_1_R) to promote insulin resistance and energy imbalance [35]. The actions of angiotensin II at AT_1_R also promote hypertension via multiple mechanisms, including vasoconstriction, sympathetic activation, arterial baroreflex impairment, the release of aldosterone, inflammation, and immune activation [36,37]. While more limited in terms of tissue expression and affinity, angiotensin II can also bind type II receptors (AT_2_R) to oppose the deleterious actions induced by AT_1_R stimulation [38]. When components of the angiotensin II axis are knocked-out globally in mice (e.g., angiotensinogen, renin, ACE, AT_1_R), these animals have increased energy expenditure and exhibit a lean phenotype [39,40,41,42]. Similarly, systemic pharmacological inhibition of angiotensin II activity with ACE inhibitors or AT_1_ receptor blockers lowers blood pressure and increases energy expenditure to lower body mass in rodents [43,44,45]. Clinically, these therapies are often used to treat hypertension in obese patients, as they have a positive metabolic profile, including modest weight loss and insulin-sensitization, in addition to their blood-pressure-lowering effects [46]. In addition to blocking angiotensin II activity, ACE inhibitors and AT_1_ receptor blockers increase the circulating levels of angiotensin-(1–7), a RAS hormone opposing angiotensin II actions [47]. Our laboratory and others have shown this endogenous angiotensin-(1–7) generation contributes to the beneficial cardiometabolic effects of these therapies in rodent models of obesity and cardiovascular disease [48,49,50].

More recently, the protective hormone angiotensin (1–7) was discovered and shown to act in opposition to the metabolic dysregulation and hypertension caused by the activation of the angiotensin II-ACE-AT_1_R axis. As shown in Figure 2, angiotensin-(1–7) is formed from angiotensin I via endopeptidases (e.g., neprilysin, prolyl oligopeptidase, thimet oligopeptidase) or from angiotensin II via ACE2 [51,52,53]. Additionally, angiotensin I can be converted by ACE2 to angiotensin-(1–9), which is then cleaved by either neprilysin or ACE to form angiotensin-(1–7). Angiotensin-(1–7) is a ligand of the Mas G-protein coupled receptor (MasR), with most data supporting that physiological actions of angiotensin-(1–7) are prevented by either MasR antagonism with A779 or genetic MasR deletion [51]. Emerging reports, however, describe potential heterodimerization and functional interactions between MasR and AT_1_R, AT_2_R, bradykinin B2, endothelin B, and dopamine D2 receptors [54,55]. In animal models and clinical populations, circulating angiotensin-(1–7) levels appear reduced with obesity, suggesting deficiency of this protective hormone [56,57,58]. Chronic restoration of angiotensin-(1–7), both peripherally and centrally, lowers blood pressure and improves insulin sensitivity, glucose tolerance, and lipid metabolism in rodent models of obesity and metabolic syndrome [52]. Additionally, two recent studies have shown that angiotensin-(1–7) administered systemically can induce adipose thermogenesis to increase energy expenditure and promote weight loss in obese mice [58,59]. Conversely, in clinical populations, genetic variants in MasR are associated with obesity risk [60]. Global MasR knockout mice also exhibit increases in blood pressure and a metabolic syndrome-like phenotype characterized by increased adiposity and impaired glucose and lipid metabolism [52].

These overall findings suggest that, in the periphery, the balance of angiotensin II versus angiotensin-(1–7) pathways is important for the regulation of metabolic and cardiovascular functions and may correlate with risk for obesity and related complications. Therapies to either decrease angiotensin II or increase angiotensin-(1–7) levels and actions systemically are effective at improving integrated cardiometabolic function in obesity, although further studies are needed to better understand precise mechanisms involved in these effects and how to translate these findings clinically, particularly for angiotensin-(1–7) pathways. Importantly, the ability of systemically administered angiotensin peptides to modulate adipose thermogenesis and energy expenditure suggests potential actions on neurocircuits controlling energy balance. Indeed, research is just beginning to explore the expression pattern and actions of RAS components on neural circuits originating in the ARC for effects on energy balance in mouse models.

## 4. Renin–Angiotensin Interactions with Arcuate Neurocircuits for Metabolic Regulation

The RAS may provide a key hormonal link between the periphery and neurocircuits controlling energy balance. Angiotensin peptides do not readily cross the blood–brain barrier but can access the central nervous system via receptors localized to blood–brain-barrier-deficient circumventricular organs, including those surrounding the ARC [61,62]. Furthermore, in disease states such as obesity and hypertension, disruptions in blood–brain-barrier permeability may provide direct access for angiotensin peptides and other circulating hormones to hypothalamic and brainstem regions involved in the control of energy balance and blood pressure [63,64]. In addition, angiotensin peptides can be formed directly within the brain via a local RAS for paracrine actions [65]. While the brain RAS has been reported to operate independently from the circulation, some reports suggest that local tissue angiotensin II generation via membrane-bound ACE requires the uptake of renin and angiotensinogen from the circulation [66]. An intracellular RAS has also been described in neurons, by which angiotensin II can be generated within cells or internalized by cells following the activation of cell surface receptors, to elicit intracrine effects via AT_1_R [65]. While the RAS is rapidly growing in complexity [37,52], this review will focus on angiotensin II, prorenin, and angiotensin-(1–7) pathways as there are currently no data regarding the role of other RAS components in the ARC related to the control of energy balance.

### 4.1. Angiotensin II Pathways

In contrast to its peripheral actions, central angiotensin II stimulates the resting metabolic rate to increase energy expenditure and promote weight loss [67]. Several components of the RAS have been described in the hypothalamus, including in the ARC, such as angiotensinogen, ACE, and ACE2 [68]. In addition, the angiotensin II AT_1_R is highly expressed in the ARC of animal models and humans [69,70,71]. A recent in silico reanalysis of published RNA-seq cell-specific gene-expression data showed RAS receptor localization in hypothalami obtained from male and female C57BL/6N mice prior to sexual maturation, precluding an examination of sex differences. These data showed that the AT_1a_R isoform is expressed in the mouse hypothalamus and selectively in a subset of AgRP neurons expressing SSt3, with no expression of the AT_1b_R isoform (Table 1) [68]. It has been hypothesized these SSt3 ARC^AgRP^ neurons project to a unique but unknown set of second-order neurons to modulate energy expenditure, whereas GABAergic ARC^AgRP^ neurons project to the PVN to participate in the control of food intake and blood pressure [72].

Consistent with this localization, Claflin et al. have shown a critical role for AT_1a_R localized to leptin receptor- and AgRP-expressing neurons in the ARC for control of thermogenic sympathetic nervous activity and resting metabolic rate (Table 2). More specifically, male and female mice with AT_1a_R deletion within leptin-expressing cells failed to show an increase in resting metabolic rate in response to HFD and deoxycorticosterone acetate-salt, with sex-specific effects not reported [67]. Importantly, the deletion of AT_1a_R in leptin-expressing cells did not alter blood-pressure control, suggesting anatomical dissociation of angiotensin II neurocircuits controlling energy balance versus blood pressure [67]. The ideal approach to selectively target central versus peripheral AT_1_R clinically is unclear but remains an active area of investigation. A subsequent report by Sapouckey et al. showed these neuronal AT_1a_R pathways appear independent of local angiotensinogen expression in both male and female mice [73]. The angiotensin II AT_2_R is present in glutamatergic POMC neurons in mouse hypothalami, but the role of these receptors remains unknown (Table 1) [68]. Finally, while not a focus of this review, angiotensin II actions in the ARC have also been implicated in the regulation of fluid balance, neuroinflammation, and reproductive functions [74,75,76,77,78].

### 4.2. Prorenin Pathways

Prorenin, an inactive precursor of renin, contains a 43-amino acid prosegment covering the active cleft and binds the prorenin receptor (PRR; Figure 2). The PRR activates prorenin in a non-proteolytic manner to increase local angiotensin II production as well as initiate angiotensin II-independent signaling [80]. The neuronal PRR appears to mediate the majority of angiotensin II formation in the central nervous system [81]. In the human brain, PRR has been shown to reside on neurons, and not astrocytes, in the hypothalamic PVN and rostral ventrolateral medulla of the brainstem [82]. This PRR immunoreactivity is significantly increased in these brain regions in hypertensive patients and correlates with systolic blood pressure but not body mass index [82]. The PRR is also highly expressed in the mouse brain including in hypothalamic and brainstem regions critical to metabolic and cardiovascular regulation [81]. A more recent study identified PRR in the ARC of male mice, with protein expression primarily co-localized with neurons [79]. Additionally, PRR gene expression has been described in both POMC (GABAergic and glutamatergic subsets) and AgRP (GABAergic and SSt3 subsets) neurons in mouse hypothalami (Table 1) [68]. A recent study by Worker et al. showed that neuron-specific PRR deletion in male mice protects against the development of HFD-induced hypertension, cardiac sympathetic overactivation, and glucose dysregulation, independent of effects on food intake or body mass (Table 2) [79]. These beneficial effects were accompanied by reduced hypothalamic Ang II levels and attenuated astrogliosis in the ARC [79]. These findings provide initial evidence that PRR mechanisms in the hypothalamus, and particularly in the ARC, contribute to HFD-induced obesity in male animal models. Additional research is needed to better understand the precise neuronal subpopulations and neural circuitry engaged by PRR in the ARC, as well as potential sex differences, but the inhibition of PRR may be a promising new approach to improve both metabolic and cardiovascular functions in obesity.

### 4.3. Angiotensin-(1–7) Pathways

Angiotensin-(1–7) levels appear reduced in obesity and metabolic syndrome, and the restoration of this hormone improves blood pressure, glucose homeostasis, and energy balance in animal models of these diseases [52]. The weight-attenuating effects of angiotensin-(1–7) are due to enhanced adipose thermogenesis and energy expenditure [58,59], suggesting the involvement of central neurocircuits. In support of the concept that angiotensin-(1–7) could modulate ARC neurocircuits for control of energy balance, the MasR is expressed in GABAergic and glutamatergic POMC neurons, as well as in GABAergic AgRP neurons in mouse hypothalami (Table 1) [68]. There are, however, no studies on the functional actions of Ang-(1–7) pathways in the ARC on energy balance (Table 2), and this is an active area of research in our laboratory. Additionally, whether ACE inhibitors or AT_1_R blockers could increase angiotensin-(1–7) levels in the ARC to alter metabolic function has not been established. Importantly, the neuronal subpopulation expression pattern for MasR differs from that of AT_1_R, supporting anatomically distinct pathways for Ang-(1–7) versus Ang II actions in the ARC. Angiotensin-(1–7) may be an attractive target for obesity given that it produces beneficial effects on metabolic and cardiovascular functions when administered either peripherally or centrally [68].

## 5. Conclusions and Future Directions

Overall, there is a critical need to better understand neural circuits controlling energy balance to develop new pharmacological strategies for obesity, which avoid the cardiovascular consequences observed with previous anti-obesity drugs. To date, approaches to increase resting metabolic rate have resulted in non-selective sympathetic overactivation to elevate blood pressure and increase cardiovascular risk (e.g., phentermine, sibutramine). These adverse cardiovascular effects are often missed at the preclinical level due to failure to consider the actions of new drugs on both metabolic and cardiovascular systems in obese animal models, prior to testing clinically. This illustrates the urgent need for investigators to conduct more integrative metabolic and cardiovascular testing for anti-obesity therapies. This review proposes that targeting the RAS may represent an ideal approach to modulate ARC neurocircuits controlling adipose thermogenesis and energy expenditure while having neutral or positive effects on the cardiovascular system. The potential targeted approaches identified include: stimulation of AT_1_R on ARC^AgRP^ neurons, inhibition of neuronal PRR, and activation of angiotensin-(1–7)-MasR pathways. These findings may reflect the dissociation of anatomical, autonomic, and signaling pathways involved in the metabolic versus cardiovascular effects of hormones, such as previously observed with angiotensin II and leptin [67,83,84].

Additional research is needed to better understand the precise intracellular signaling mechanisms engaged by RAS pathways within the ARC, as well as to explore the role of additional RAS components on ARC neurocircuits controlling energy balance. Furthermore, some of the studies cited in this review were conducted only in male rodent models or did not report outcomes by sex. There are well-established sex differences in obesity in both animal models and clinical populations, with premenopausal females generally being protected from obesity-related metabolic and cardiovascular derangements despite having higher adiposity and obesity prevalence. This protection may, in part, reflect sex differences in the ARC neurocircuits controlling energy balance. Female mice have more ARC POMC neurons, with these neurons displaying enhanced excitability compared with males [85]. This enhanced excitability has been, in part, attributed to estrogen actions on estrogen receptor-α receptors localized to ARC^POMC^ neurons [86]. In addition, females are more responsive to pharmacologically induced WAT browning due to greater levels of estrogen-dependent sympathetic innervation [87]. Thus, obese females may be more sensitive to the activation of ARC^POMC^-sympathoexcitatory pathways controlling adipose thermogenesis and energy expenditure. While there are no data available for RAS actions on ARC neurocircuits, sex differences have been reported for circulating angiotensin peptides in obesity, as well as for glycemic responses to systemic angiotensin-(1–7) treatment [88,89,90,91]. A better understanding of the mechanisms by which the RAS interacts with the ARC to control energy balance versus blood pressure will hopefully lead to additional advances in the quest to find novel and more selective anti-obesity therapies.

## Figures and Tables

**Table 1:** Localization of Renin–Angiotensin Receptors on Neuronal Subpopulations Controlling Energy Balance in the Mouse Hypothalamus.

RAS Receptor	Neuronal Subpopulations *
AT_1a_R	AgRP (Sst3)
AT_1b_R	No expression
AT_2_R	POMC (vGlut2 11)
PRR	POMC (GABA 7), POMC (vGlut2 11), AgRP (Sst3), AgRP (GABA 14)
MasR	POMC (GABA 7), POMC (vGlut2 11), AgRP (GABA 14)

* Data obtained from a recent in silico reanalysis of published RNA-seq cell-specific gene-expression data in hypothalami obtained from male and female C57BL/6N mice during postnatal days 14–28, prior to sexual maturation [68]. RAS, renin–angiotensin system; AT_1_R, angiotensin II type I receptors; AT_2_R, angiotensin II type II receptors; PRR, prorenin receptor; MasR, angiotensin-(1–7) *mas* receptors; ARC, arcuate nucleus of the hypothalamus; POMC, proopiomelanocortin; AgRP, agouti-related peptide; vGlut, vesicular glutamate transporter; GABA, γ-aminobutyric acid; Sst3, somatostatin-receptor subtype 3.

**Table 2:** Functional Roles of Renin–Angiotensin Receptors in the Hypothalamic Arcuate Nucleus in Metabolic Regulation.

RAS Receptor	Functions	(Ref.) Species, Sex
AT_1a_R	Increase resting metabolic rate; no effect on blood pressure	[67,73] Mice, Male and Female
AT_1b_R	Unknown	
AT_2_R	Unknown	
PRR	Increase blood pressure and impair glycemic control; no effect on body weight or food intake	[79] Mice, Male
MasR	Unknown	

RAS, renin–angiotensin system; AT_1_R, angiotensin II type I receptors; AT_2_R, angiotensin II type II receptors; PRR, prorenin receptor; MasR, angiotensin-(1–7) *mas* receptors.

## Data Availability

Not applicable.
